# Supplementary figures and images for: Effective degradation of various bacterial toxins using ozone ultrafine bubble water
Source: PLoS One. 2024 Jul 10;19(7):e0306998. doi: 10.1371/journal.pone.0306998 (PMC11236168; doi:10.1371/journal.pone.0306998)

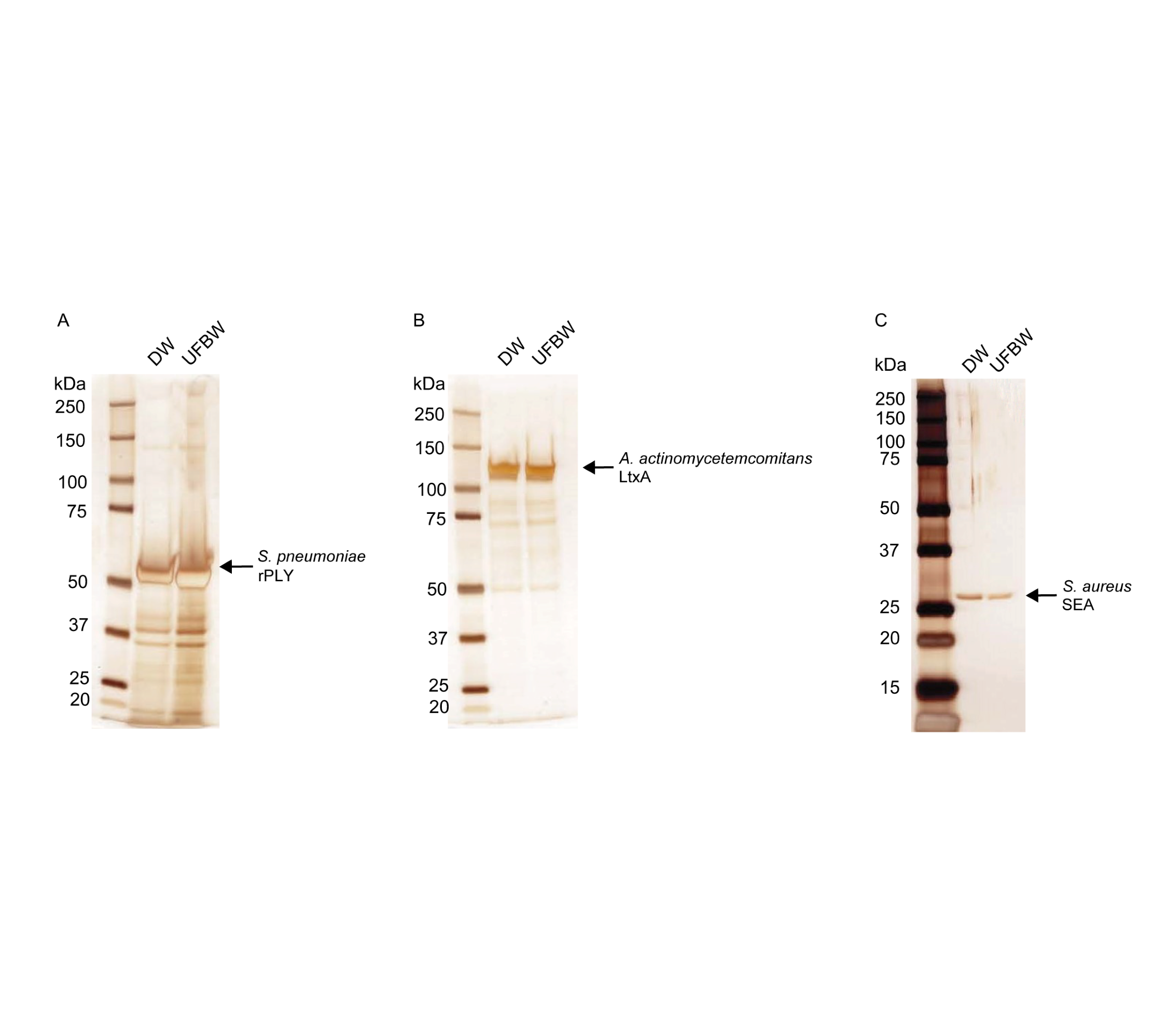

Supplement: S1 Fig — (A) Recombinant penumolysin, (B) Aggregatibacter actinomycetemcomitans leukotoxin, and (C) purified Staphylococcus enterotoxin A were added to UFBW or distilled water, followed by SDS-PAGE and silver staining. (TIF) [file pone.0306998.s001.tif]

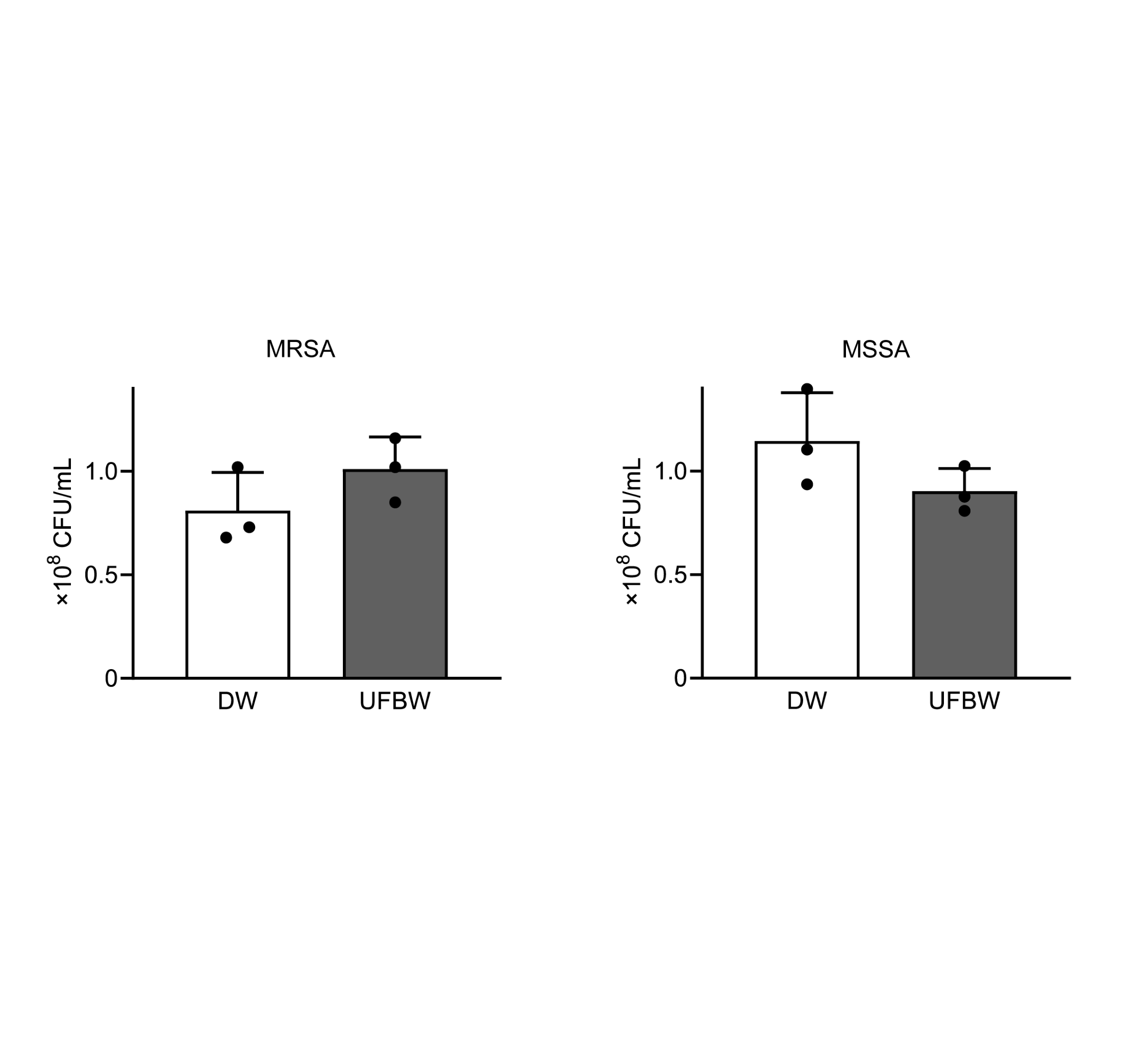

Supplement: S2 Fig — Methicillin-resistant S. aureus strain (MRSA) NILS2 and methicillin-susceptible S. aureus strain (MSSA) NILS6 were exposed to UFBW or distilled water (DW) for 1 min. The bacterial loads of S. aureus NILS2 and NILS6 were determined using colony counting. Data are presented as the mean ± SD of triplicate experiments and were evaluated using a one-way analysis of variance with a Student’s t-test. *P < 0.05. N = 3 for each bacterial strain. ND was undetected or below the detection limit (< 105 CFU/mL). (TIF) [file pone.0306998.s002.tif]

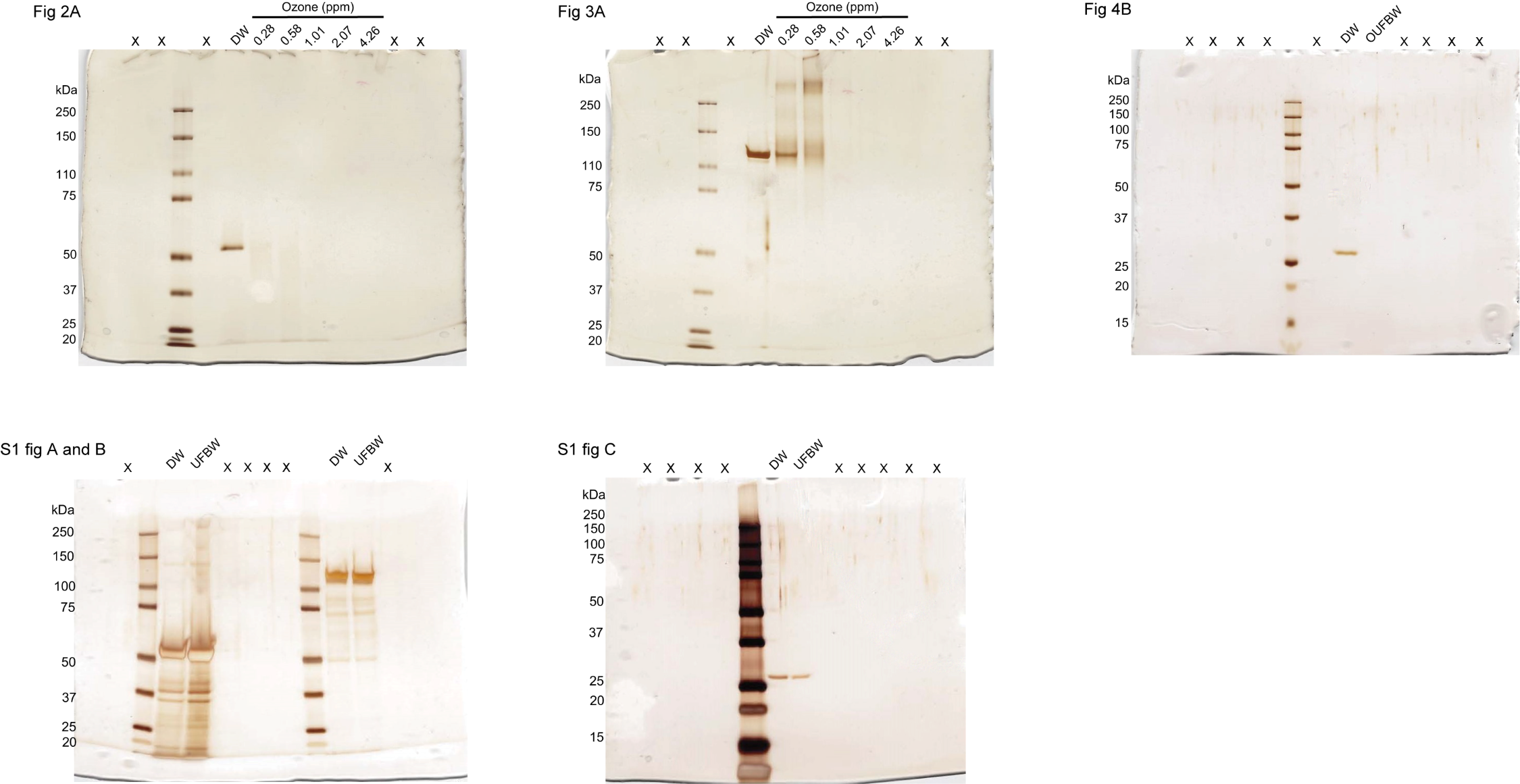

Supplement: S3 Fig — Unprocessed silver-stained images are shown in each figure. Lanes not induced in the final figure marked with an “X” above the lane. Images were obtained by scanning the gel using an image scanner. (TIF) [file pone.0306998.s003.tif]
