# Supplementary material for: Effective degradation of various bacterial toxins using ozone ultrafine bubble water
Source: PLoS One. 2024 Jul 10;19(7):e0306998. doi: 10.1371/journal.pone.0306998 (PMC11236168; doi:10.1371/journal.pone.0306998)
Supplement: Attachment — Submitted filename: Response to reviewers.docx [file pone.0306998.s005.docx]

**Response to Editor and Reviewers**

**Response to Editor**

We thank the editor for their critical comments that have helped us to improve our manuscript. As indicated in the responses below, we have considered all these comments and addressed each of them during our revision of the manuscript.

**<Comment #1>** Please ensure that your manuscript meets PLOS ONE's style requirements, including those for file naming.

**<Response>** According to the editor’s comment, we have ensured that our manuscript and figures adhere to PLOS ONE's style requirements and have renamed the file accordingly.

**<Comment #2>** Please note that funding information should not appear in any section or other areas of your manuscript. We will only publish funding information present in the Funding Statement section of the online submission form. Please remove any funding-related text from the manuscript.

**<Response>** Following the editor’s comment, we have removed all funding-related text from the revised manuscript.

**<Comment #3>** We note that the grant information you provided in the ‘Funding Information’ and ‘Financial Disclosure’ sections do not match. When you resubmit, please ensure that you provide the correct grant numbers for the awards you received for your study in the ‘Funding Information’ section.

**<Response>** At the time of resubmission, the ‘Funding Information’, ‘Financial Disclosure’, and 'Grant numbers' have been revised to match their descriptions. We have described the revised Financial Disclosure statement in cover letter.

**<Comment #4>** Please confirm at this time whether or not your submission contains all raw data required to replicate the results of your study.

**<Response>** Lines 503 and Table S1 (in the revised version): According to the editor’s suggestion, we submitted raw data files as the values used to build graphs for Figures 2B, 2C, 3B, 4A, 5A, 5B and Figure S2.

**<Comment #5>** Please include your full ethics statement in the ‘Methods’ section of your manuscript file. In your statement, please include the full name of the IRB or ethics committee who approved or waived your study, as well as whether or not you obtained informed written or verbal consent. If consent was waived for your study, please include this information in your statement as well.

**<Response>** Lines 120, 121-122 (in the revised version): Following the editor’s comment, we added a description of the full name of the IRB or ethics committee in line 120 (in the revised version). Additionally, we described written informed consent of this study in lines 121-122 (in the revised version) as follows: “Written informed consent was obtained from all donors in the presence of a third party before participating in the study (permit # 2018-0075).”

**<Comment #6>** Please ensure that your Figures adhere fully to journal guidelines and provide the original underlying images for all blot or gel data reported in your submission.

**<Response>** Lines 500-502 and S3 Figure (in the revised version): According to the editor’s comment and journal guidelines, we submitted raw images of silver stain gels of Figure 2A, 3A, 4B, Figure S1A, S1B, and S1C.

**Response to Reviewer 1**

We are grateful to Reviewer 1 for the critical comments and suggestions, which have helped us improve our paper considerably. As indicated in the following responses, we have considered all of these comments and suggestions in the revised version of our paper.

**<Comment #1>** Draft need writing improvement by native English speaker.

**<Response>** Based on the reviewer’s suggestion, the revised manuscript has been proofread by a native English speaker. The manuscript was revised according to the proofreading. The revised sections are highlighted. Additionally, we have submitted an English proofreading certificate for this manuscript.

**<Comment #2>** In introduction section, author should mention how ozone acts antimicrobial i.e mechanism through which O3 kills microbes.

**<Comment #3>** Author discuss and showed the anti-microbial effect of Ozone on bacterial cells, whether Ozone also have anti-viral properties should be discussed in draft

**<Response to comment #2 and #3>** Lines 62-65 (in the revised version): Following the reviewer comment, we added a description of the antimicrobial mechanism of ozone as follows: “Ozone disrupts the integrity of the bacterial cell envelope through oxidation of the phospholipids and lipoproteins. In addition, ozone inhibits fungal cell growth. In the case of viruses, ozone has been reported to damage the viral capsid and disrupt the reproductive cycle by interfering with viral cell contact through peroxidation.”

The targets of this study were bacteria and bacterial toxins. Therefore, we did not analyze the effects of ozone ultrafine bubble water on the viruses.

**<Comment #4>** Author should also discuss or highlight the limitations which restricts use of ozone as antimicrobial agents

**<Response>** Lines 309-318 (in the revised version): According to the reviewer’s suggestion, we added a paragraph in the discussion section about the limitations that restrict the use of ozone as an antimicrobial agent in lines 309-318 as follows: “Previous studies indicated ozone gas and ozonated water exert antimicrobial activity against various microorganisms such as bacteria, fungi, and virus. In the present study, OUFBW (0.8–4 ppm of ozone concentration) sterilized planktonic *S. aureus*, including antibiotic-resistant strains. However, in our previous study, OUFBW containing approximately 5 ppm ozone was unable to completely sterilize bacteria adhered to toothbrushes and gauze. Shichiri-Negoro *et al*. reported that treatment with OUFBW (9 or 11 ppm ozone) reduced the viable counts of *Candida albicans* in biofilms. However, biofilms that formed within 24 h were not completely removed. These results indicate that OUFBW could not sterilize bacteria and fungi in a complicated structure. To exert antimicrobial properties, microorganisms need to be directly exposed to OUFB.”

With the addition of the description, we have added references and changed the reference order.

**Response to Reviewer 2**

We thank Reviewer 2 for their critical comments and suggestions, which have helped us improve our paper considerably. According to your comments, we have performed some additional experiments. And then, we added a supporting Figure in response to your comment. As indicated in the following responses, we have considered the comments and suggestions in the revised manuscript.

**<Comment #1>** In all the experimental groups, a proper control is missing. Authors are requested to use UFBW and OUFBW and repeat the experiment for Figure 2A, 3A and 4B i.e. degradation of rPLY, LtxA and SEA. It should justify the overall findings. Author can add the new result as supplementary or main Figure and discuss it in main texts.

**<Response>** Figures S1, S2 and lines 86, 92, 93, 101, 106, 150, 151, 169, 179, 180, 204, 205, 206-208, 228, 231, 233, 234, 237, 238 and 489-503 (in the revised version): We agree with the reviewer’s comments, and we performed additional experiments about whether ultrafine bubble water exerts bactericidal activity and degrade rPLY, LtxA and SEA. Ultrafine bubble water showed no bactericidal activity against *Staphylococcus aureus* and did not decompose bacterial toxins. Thank you for your comment. We have added Figure S1 and S2 to the Supporting Information. We have also added Figure legend for these Figures in lines 489-499 as follows: “**S1 Fig. Ultrafine bubble water (UFBW) does not degrade bacterial toxins.** (A) Recombinant penumolysin, (B) *Aggregatibacter actinomycetemcomitans* leukotoxin, and (C) purified Staphylococcus enterotoxin A were added to UFBW or distilled water, followed by SDS-PAGE and silver staining. **S2 Fig. Ultrafine bubble water (UFBW) does not induce a bactericidal effect against *Staphylococcus aureus*.** Methicillin-resistant *S. aureus* strain (MRSA) NILS2 and methicillin-susceptible *S. aureus* strain (MSSA) NILS6 were exposed to UFBW or distilled water (DW) for 1 min. The bacterial loads of *S. aureus* NILS2 and NILS6 were determined using colony counting. Data are presented as the mean ± SD of triplicate experiments and were evaluated using one-way analysis of variance with a Student’s t*-*test. **P* < 0.05. N = 3 for each bacterial strain. ND stands for undetected and indicates below the detection limit (< 10^5^ CFU/mL).”

With the addition of the supporting information, we have added descriptions to the Materials and Methods and Results sections of the revised manuscript in lines 86, 92, 93, 101, 106, 150, 151, 169, 179, 180, 204, 205, 206-208, 228, 231, 233, 234, 237, and 238.

**<Comment #2>** Authors have used 3 healthy donors blood samples and have performed the experiment on erythrocytes and neutrophils. In the given results, Figure 2B, 2D and 3B the N is different (N is 4 and 5).

**<Comment #6>** In material and method: line 111(in the revised version): please mention if the human cells were freshly/immediately used for the experiment after blood draw or they (3 donor cells) were frozen after blood draw and then used together for the experiment.

**<Response to comments #2 and #6>**　Lines 123 and 124 (in the revised version): According to the reviewer’s suggestion, we have added a description to the revised manuscript as follows: “Human blood was obtained from one donor per experiment and the human cells were used for the experiment immediately after blood draw.”

**<Comment #3>** Please mention N for all the sub-Figures in the Figure legend.

**<Response>**　Lines 194, 200, 222, 245, 269, and 498 (in the revised version): According to reviewer’s suggestion, we have added a description of N to the figure legend of the revised manuscript in lines 194, 200, 222, 245, and 269.

**<Comment #4>** Figure 2C and 3C, authors have shown the damage to neutrophils ‘cell walls’. Please correct it with ‘cell membrane’ as the mammalian cells do not have cell wall. Authors can quantify the membrane damage and present it with statistical bar diagram. **<Response>** Lines 184 and 212 (in the revised version): According to the reviewer’s suggestion, we have corrected ‘cell walls’ with ‘cell membrane’ in lines 184 and 212 (in the revised version). Although we attempted to quantify neutrophil cell membrane damage by 7-AAD and cytocalcein violet 450 (ab176749, Abcam, Cambridge, UK) staining, this was difficult because the dead cells were almost completely destroyed and removed by washing. Representative microscopic images are shown in Figures 2C and 3C.

**<Comment #5>** In the abstract section: line 30, it’s a repetition of sentence ‘high concentration of ozone gas’. Kindly correct it.

**<Response>** The Abstract has been revised accordingly. We apologize for the error.
