# Supplementary material for: Effective degradation of various bacterial toxins using ozone ultrafine bubble water
Source: PLoS One. 2024 Jul 10;19(7):e0306998. doi: 10.1371/journal.pone.0306998 (PMC11236168; doi:10.1371/journal.pone.0306998)
Supplement: Attachment — Submitted filename: Response to Reviewers.docx [file pone.0306998.s006.docx]

**Response to Editor and Reviewer #2**

We thank the editor and all reviewers for their critical reviews. As indicated in the responses below, we have considered all your comments and addressed each of them during our R2-version of the manuscript.

**<Reviewer #2 Comment #1>** However, the second comment from the previous request was not addressed which is critically important to clarify the technicality of the experiment. “Authors have used 3 healthy donors blood samples and have performed the experiment on erythrocytes and neutrophils. In the given results, Figure 2B, 2D and 3B the N is different (N is 4 and 5). Authors have used biological replicates or technical replicates. Kindly clarify and mention in the legend.” This is important to clarify how N=3 (healthy donor) became N=4 or N=5 in the results.

**<Response #1>** Lines 113-114, 123-124, 196, 203, 225-226 in the R2-version: According to the reviewer’s comment, we have added a description to the revised manuscript as follows, respectively: “Lines 113-114: Heparinized whole blood samples were obtained from three healthy donors (A, B and C) between April 17, 2023, and July 26, 2023.”, “Lines 123-124: Human blood was obtained from one donor per experiment (Fig 2B; N = 5 wells from donor A, Fig 2D; N = 4 wells from donor B, Fig 3B; N = 5 wells from donor C), and human cells were used for the experiment immediately after blood collection.”, “Line 196 (Fig. 2B): N = 5 wells per each group (from one healthy donor). “, “Line 203 (Fig. 2D): N = 4 wells per each group (from one health donor).”, “Lines 225-226 (Fig. 3B): N = 5 wells per each group (from one healthy donor).”

**<Reviewer #2 Comment #2>** Cell membrane damage quantification (line 184 and 203 in revised draft): Without a clear marker or proper quantification, it is difficult to conclude the membrane damage only by microscopic observation. These cells may change their granularity and phenotype in response to the toxin and may look like having damaged membrane. Authors are advised to modify the statements as ‘appears damaged the cell membrane/ appears damage the human neutrophil’.

**<Response #2>** Lines 185-187 and 215-216 in the R2-version: In accordance with the reviewer #2’s comment, we have modified the text that was pointed out as follows: “Lines 185-187: Microscopic examination revealed that although treatment with rPLY appears damaged the cell membranes of neutrophils, OUFB-RPMI-treated rPLY did not induce any morphological changes in human neutrophils (Fig 2C).” and “Lines 215-216: Although LtxA appears damaged the neutrophil cell membranes, OUFB-RPMI-treated LtxA did not induce morphological changes in human neutrophils (Fig 3C).”

**<Journal Requirements>** Please review your reference list to ensure that it is complete and correct. If you have cited papers that have been retracted, please include the rationale for doing so in the manuscript text, or remove these references and replace them with relevant current references. Any changes to the reference list should be mentioned in the rebuttal letter that accompanies your revised manuscript. If you need to cite a retracted article, indicate the article’s retracted status in the References list and also include a citation and full reference for the retraction notice.

**<Response>** Lines 337-493 in the R2-version: In accordance with the 'Journal Requirements', we have reviewed and revised the reference list.
